# A Mechanistic Study of the Association Between Symbolic Approximate Arithmetic Performance and Basic Number Magnitude Processing Based on Task Difficulty

**DOI:** 10.3389/fpsyg.2018.01551

**Published:** 2018-09-11

**Authors:** Wei Wei, Wanying Deng, Chen Chen, Jie He, Jike Qin, Yulia Kovas

**Affiliations:** ^1^Department of Psychology and Behavioral Sciences, Zhejiang University, Hangzhou, China; ^2^Department of Psychology, The Ohio State University, Columbus, OH, United States; ^3^Department of Psychology, Goldsmiths, University of London, London, United Kingdom; ^4^Laboratory for Cognitive Investigations and Behavioural Genetics, Tomsk State University, Tomsk, Russia

**Keywords:** symbolic approximate arithmetic, kindergartner, number processing, number line estimation, number comparison, task difficulty

## Abstract

Two types of number magnitude processing – semantic and spatial – are significantly correlated with children’s arithmetic performance. However, it remains unclear whether these abilities are independent predictors of symbolic approximate arithmetic performance. The current study addressed this question by assessing 86 kindergartners (mean age of 5 years and 7 months) on semantic number processing (number comparison task), spatial number processing (number line estimation task), and symbolic approximate arithmetic performance with different levels of difficulty. The results showed that performance on both tasks of number magnitude processing was significantly correlated with symbolic approximate arithmetic performance, but the strength of these correlations was moderated by the difficulty level of the arithmetic task. The simple symbolic approximate arithmetic task was equally related to both tasks. In contrast, for more difficult symbolic approximate arithmetic tasks, the contribution of number comparison ability was smaller than that of the number line estimation ability. These results indicate that the strength of contribution of the different types of numerical processing depends on the difficulty of the symbolic approximate arithmetic task.

## Introduction

Arithmetic competency is an important aspect of mathematical ability. Over the past few decades, many studies have investigated the cognitive mechanisms underlying exact arithmetic ability (De Smedt et al., 2013; Moeller et al., 2015; see Arsalidou and Taylor, 2011; Schneider et al., 2017, for reviews). However, less is known about the cognitive mechanisms underlying symbolic approximate arithmetic calculations, such as solving the following task: “give an approximate answer for 38 × 21 in 5 s.”

Symbolic approximate arithmetic performance refers to the ability to provide an approximate answer rather than an exact one (Gilmore et al., 2007; McNeil et al., 2011; Xenidou-Dervou et al., 2015). This ability plays an important role in mathematical learning (Xenidou-Dervou et al., 2013). This importance has begun to receive recognition by educational authorities. For example, symbolic approximate arithmetic performance is listed as an important part of mathematical learning by The National Council of Supervisors of Mathematics and the National Council of Teachers of Mathematics in the United States (1989), as well as by the Ministry of Education in Japan (1989). Understanding cognitive mechanisms underlying symbolic approximate arithmetic performance will help with the designing of curricula that develop symbolic approximate arithmetic skills.

Recent research has begun to provide insights into these mechanisms. Research has suggested that, unlike exact arithmetic ability, symbolic approximate arithmetic performance may not be influenced by culture (Reys and Yang, 1998), language, or education (Spelke and Tsivkin, 2001; Nys et al., 2013). For example, recent research has found that preschool children can solve symbolic approximate arithmetic problems with large numbers, even if they cannot provide exact answers (Gilmore et al., 2007; McNeil et al., 2011; Xenidou-Dervou et al., 2015).

Two types of tasks have typically been used to assess basic numerical magnitude processing: *the number magnitude comparison task*, primarily tapping into number semantic processing (Pinel et al., 2001; Rousselle and Noël, 2007; Holloway and Ansari, 2009; see De Smedt et al., 2013, for a review) and *the number line estimation task*, primarily tapping into spatial number processing (Dehaene et al., 2003; Hubbard et al., 2005; Siegler and Ramani, 2008; Berteletti et al., 2012; see Moeller et al., 2015, for a review). These abilities are commonly referred to in the literature as *number sense*, although recent research suggests that it is a highly heterogeneous concept (e.g., Berch, 2005; Halberda et al., 2008; Tosto et al., 2017; see Cohen Kadosh et al., 2008; De Smedt et al., 2013, for reviews).

Number semantic processing and number spatial processing are both correlated with exact arithmetic processing. For example, correlations between exact arithmetic processing and number semantic processing have been found in typically developing children (Durand et al., 2005; Bartelet et al., 2014; Vanbinst et al., 2015), in children with developmental dyscalculia (Landerl et al., 2004; Mussolin et al., 2010), as well as in training studies (Wilson et al., 2006, 2009). Similarly, correlations between exact arithmetic processing and number spatial processing have been shown in typically developing children (Booth and Siegler, 2008; Laski and Yu, 2014), as well as in training studies (Siegler and Ramani, 2008; Kucian et al., 2011).

It is possible that number semantic processing and number spatial processing may relate to arithmetic abilities through a common mechanism. For example, Laski and Siegler (2007) examined the performance on number line estimation and number comparison tasks in 5–8-year-old children and observed strong associations between the two tasks within each grade. However, other studies suggest that the two abilities influence arithmetic performance through different mechanisms, as the two are at least partially independent. For example, Sasanguie and Reynvoet (2013) found that children in grades 1–3 who were faster at comparing numbers performed better on a timed arithmetic test 1 year later. In contrast, no significant associations were found between performance on symbolic number line estimation task and a timed arithmetic test. Recent data provided by Linsen et al. (2014) further showed significant associations between number processing (including number comparison and number line estimation tasks) and the more specific mathematical skill of mental subtraction. In their study, the association between number comparison and mental subtraction remained after controlling for the number line estimation, whereas the association between number line estimation and mental subtraction disappeared after controlling for the number comparison task.

Both number magnitude comparison ability (Gilmore et al., 2007) and number line estimation ability (Gunderson et al., 2012) have been found to be associated with children’s performance on symbolic approximate arithmetic tasks. However, most previous studies have examined only one of these basic numerical processing tasks at a time, which makes it difficult to evaluate the extent to which they differentially predict symbolic approximate arithmetic performance.

It is necessary to involve two number magnitude processing tasks in one study to investigate their differential influence on symbolic approximate arithmetic performance. We put forward our first hypothesis, “*Number semantic processing and spatial processing are significantly correlated with the performance of symbolic approximate arithmetic tasks*.”

It is possible that relations between number magnitude processing and arithmetic processing are moderated by the difficulty of the arithmetic task, in that different difficulty levels of arithmetic problems rely on semantic and spatial number tasks to a different extent. For example, a correlation between performance on exact arithmetic processing and number semantic processing has been observed in simple exact arithmetic tasks (e.g., single-digit arithmetic problems) (Landerl et al., 2004; Durand et al., 2005; Bugden et al., 2012; Bartelet et al., 2014; Vanbinst et al., 2015). In contrast, other studies found a correlation between exact arithmetic processing and number spatial processing, which has been observed in difficult arithmetic tasks, such as two-digit or three-digit arithmetic problems. Complex mathematical problems are more dependent on spatial processing when compared with simple problems. For the simple arithmetic problems, participants retrieved the answers from long-term semantic working memory (Geary et al., 1996; LeFevre et al., 1996; Delazer and Benke, 1997; McLean and Hitch, 1999), whereas much more visuospatial processing was involved in the processing of complex arithmetic problems (Zago et al., 2001; Berteletti et al., 2015).

It is unclear if the symbolic approximate arithmetic task has the same effects as the exact arithmetic task. We put forward our second hypothesis, “*The relations between number semantic processing and spatial processing and symbolic approximate arithmetic ability vary as a function of the difficulty of the symbolic approximate arithmetic tasks*.”

## Materials and Methods

### Participants

A total of 94 typically developing children from middle-to-high socioeconomic status (SES) backgrounds were recruited from three kindergartens in the urban area of Hangzhou, China. Data of eight children were removed from the analyses because they either correctly answered at least one question in the probe stage (see Procedure for details) of the symbolic approximate arithmetic task (*n* = 5) or did not complete all the tasks (*n* = 3). The final sample included 86 children (45 boys and 41 girls). Their mean age was 5 years and 7 months (ranging from 5 years and 1 month to 6 years and 3 months). Similar to most countries, formal mathematics education starts in the first year of elementary school in China; therefore, children in this study were assessed prior to receiving formal mathematical instruction. Permission to conduct the study was given by the principals of the kindergartens. Written informed consents were obtained from all the parents. The study was approved by the principals of the kindergartens and the ethics committee at the Zhejiang University, China.

### Measures

#### Symbolic Approximate Arithmetic Task

The symbolic approximate arithmetic task was adapted from Gilmore et al. (2007). Arithmetical questions were presented both visually on a computer screen and verbally by the experimenter. The children had to indicate which side of the screen had a larger numerical magnitude through mental arithmetic. For example, on the screen, one cartoon character first received a bag of candies marked with the number 13 and then received a second bag marked with the number 22. Another cartoon character received a bag of candies marked with the number 28. The children needed to determine which character had more candies in total. Each trial would remain on the screen until the participants responded. This task consisted of 5 practice problems and 24 formal problems. The formal problems were divided into three levels of difficulty according to the ratios of the sum of the problem to the comparison number, that is, 4:7 (Level 1, the easiest level), 4:6 (Level 2, the medium level), and 4:5 (Level 3, the hardest level). The numbers ranged from 6 to 56. The exact answer was larger than the comparison number in half of the problems, whereas it was smaller in the other half of the problems. The formal problems part was split into three blocks. Each block included eight problems. The difficulty levels varied within each block. Error rate was used as the index of performance. There was no time limitation for the children’s responses. Spearman–Brown corrected split half reliability was *r* = 0.76.

#### Symbolic Number Comparison Task

The symbolic number comparison task was adapted from Gilmore et al.’s (2007) study. In the number comparison task, two two-digit numbers were used. The numbers were presented on a computer screen at the same time, and the children were asked to judge which number was larger. A total of 5 practice problems were followed by 24 formal problems. The children were required to make the judgment. If the children chose the left number, they pressed the “F” key on the computer keyboard; if they chose the right number, they pressed the “J” key. Each trial would remain on the screen until the participants responded. The order of the presentation was random for each participant. After each practice trial, the children would see a smiling face on the screen if they responded correctly or a crying face if they responded incorrectly. Only children with accuracy above 60% in the practice problems would be given the formal problems. No feedback was given following the formal trials. The index of performance was the error rate. Spearman–Brown corrected split half reliability was *r* = 0.83.

#### Number Line Estimation Task

This task was adopted from Siegler and Opfer’s (2003) study. The children were given 28 sheets of paper, 2 for practice trials and 26 for formal trials, each with the same 25 cm number line printed in the center and a number between 0 and 100 printed 2 cm above the middle of the line. The experimenter initially told the children the following: “Each number has its own specific position on the number line and you should mark the position where you think the number actually is on the line using a pencil. Try your best to do it exactly.” For two practice problems, the children were asked to mark the location of the number 50. If they failed, the experimenter would help them to find the correct location. The formal problems had “0” written below the start of the number line and “100” written below the end point. A total of 26 trials were held, respectively, for the 26 numbers to be estimated. The numbers used in the experiment (3, 4, 6, 8, 12, 14, 17, 18, 21, 24, 25, 29, 33, 39, 42, 48, 52, 57, 61, 64, 72, 79, 81, 84, 90, and 96) were taken from Booth and Siegler’s (2006) study. The order was presented randomly for each child. The main performance index was the percent of absolute error [PAE = (|estimate-estimated quantity|/scale of estimates) × 100], where estimate is the participant’s answer, estimated quantity is the correct answer, scale of estimates is 100 in the current study. PAE reflects the accuracy of numerical estimation and has been used in a large number of studies (Booth and Siegler, 2008; Laski and Yu, 2014; Xenidou-Dervou et al., 2015). A smaller PAE indicates more accurate numerical estimation. Spearman–Brown corrected split half reliability was *r* = 0.81.

### Procedure

The symbolic approximate arithmetic and number comparison tasks were presented on a laptop with a 15-inch monitor. The stimuli for the symbolic approximate arithmetic and number comparison tasks were presented using Presentation^®^ software (version 0.71; Neurobehavioral Systems, Berkeley, CA, United States). The number line estimation was a paper-and-pencil task.

For all experimental measures, the children were tested one by one in a quiet room in the kindergarten, accompanied by an experimenter. The children performed the tasks in the following order: the symbolic number comparison task, the number line estimation task, and the approximate addition arithmetic task. A short break of about 2 min was provided between each task.

In order to prevent the children from performing exact calculations in the symbolic approximate arithmetic task, a probe stage was then conducted. In the probe stage, the children were asked to provide the exact answers for two problems, which were chosen randomly from the ones they had performed correctly in the formal part of the symbolic approximate arithmetic task. The data of the children who correctly answered at least one question in the probe stage were removed from the analysis. This approach ensured that the participants were unable to perform the exact calculations.

The whole test took approximately 25 min for each child. Following the experiment, each child received a sticker as a reward.

### Data Analysis

The following data analysis was performed using the SPSS 19.0 software (SPSS Inc., Chicago, IL, United States). Analyses were performed on error rate for the symbolic approximate arithmetic and symbolic number comparison tasks and on PAE for the number line estimation task. No participants were outliers (three *SD* above or below the group mean) for each task. Error rates and PAE have the same direction. First, we calculated the correlation coefficients between the symbolic approximate arithmetic task and the two basic number processing tasks after controlling for gender and age. To explore the specific roles of the number comparison and number line estimation abilities in different levels of symbolic approximate arithmetic performance, we conducted hierarchical regression analyses.

## Results

### Descriptive and Preliminary Analysis

All dependent measures and predictors are presented in **Table 1**. The correlations between the different levels of symbolic approximate arithmetic task and basic numerical magnitude processing task are presented in **Table 2** (controlling for gender and age). First, a series of analyses were conducted in order to verify that our children were able to perform the tasks. The error rate of approximate addition showed that children performed below chance level (50%) on all three levels (Level 1: *M* = 28%, *t*_85_ = -11.060, *p* < 0.001; Level 2: *M* = 33%, *t*_85_ = -7.421, *p* < 0.001; and Level 3: *M* = 38%, *t*_85_ = -5.881, *p* < 0.001). These results were similar to Gilmore et al.’s (2007) study, which had a 26.7% error rate for approximate addition problems. The children’s error rate for number comparison tasks was also below 50% (*M* = 22%, *t*_85_ = -13.036, *p* < 0.001), which was similar to Gilmore et al.’s (2007) study (19.6% error rate for number comparison tasks). The children’s mean PAE was 20.65%, which was similar to the previous studies (Booth and Siegler, 2006, *M* = 24%).

**Table 1:** Descriptive statistics of kindergartners’ performance on measures of symbolic approximate arithmetic ability, symbolic number comparison ability, and number line estimation ability.

Task	Index	Mean (*SD*)	Range
Symbolic approximate arithmetic	Error rate	0.33 (0.16)	0.00 ∼ 0.58
Level 1 (4:7)	Error rate	0.28 (0.19)	0.00 ∼ 0.62
Level 2 (4:6)	Error rate	0.33 (0.21)	0.00 ∼ 0.75
Level 3 (4:5)	Error rate	0.38 (0.19)	0.00 ∼ 0.88
Symbolic number comparison	Error rate	0.22 (0.20)	0.00 ∼ 0.62
Number line estimation	PAE	20.65% (8.55)	3.68% ∼ 41.16%

*PAE = percent of absolute error*.

**Table 2:** Correlations between the basic numerical magnitude processing tasks and different difficulty levels of the symbolic approximate arithmetic tasks after controlling for gender and age.

	1	2	3	4	5
**Symbolic approximate arithmetic**					
(1) Level 1	–				
(2) Level 2	0.59***	–			
(3) Level 3	0.42***	0.48***	–		
(4) All Levels	0.81***	0.86***	0.77***	–	
**Basic numerical processing**					
(5) Number comparison	0.33**	0.40***	0.35**	0.44***	–
(6) Number line estimation	0.29**	0.39***	0.39***	0.44***	0.44***

*^∗∗^*p* < 0.01 and ^∗∗∗^*p* < 0.001. “All Levels” was the composite score of Level 1, Level 2, and Level 3*.

In addition, a repeated measure ANOVA was conducted to test the effect of difficulty on symbolic approximate arithmetic performance, *F*_(2,170)_ = 13.263, *p* < 0.001. The *post hoc* results showed that as the difficulty increased, the accuracy of symbolic approximate arithmetic performance decreased. Error rate for Level 1 of symbolic approximate arithmetic performance was significantly lower than that for Level 3 [*F*_(1,85)_ = 24.638, *p* < 0.001], Level 1 was significantly lower than Level 2 [*F*_(1,85)_ = 9.328, *p* = 0.003], and Level 2 was significantly lower than Level 3 [*F*_(1,85)_ = 5.012, *p* = 0.028].

### Hierarchical Regression Analysis

Two models of hierarchical regression analysis were carried out to further examine the relationships among number comparison performance, number line estimation performance, and different levels of symbolic approximate arithmetic performance. The error rates of the three different levels of symbolic approximate arithmetic performance were the outcome variables.

The first regression model tested whether number magnitude comparison ability was associated with different levels of symbolic approximate arithmetic performance after controlling for gender, age, and number line estimation performance. Gender and age were entered into the model first, following which the PAE of number line estimation performance and the error rate of number comparison performance were entered, respectively. For Level 1 and Level 2, number comparison performance was a significant predictor of symbolic approximate arithmetic performance after controlling for gender, age, and number line estimation performance. However, for the most difficult level (Level 3), number comparison performance was not a significant predictor. For Level 1, number line estimation performance was not a significant predictor for symbolic approximate arithmetic performance after number comparison performance was entered into Model 1. However, for Levels 2 and 3, number line estimation performance continued to be a significant predictor of symbolic approximate arithmetic performance, even when number comparison performance was entered into the model.

The *R*-square change carried by number comparison performance decreased, becoming 4.2% for Level 1, 4.9% for Level 2, and 3.0% for Level 3, after controlling for number line estimation performance, gender, and age. For difficulty Levels 1 and 2, number comparison performance significantly improved the fit of the model, whereas for Level 3, it did not significantly improve the fit of the model (**Table 3**, Model 1).

**Table 3:** Hierarchical regression analysis predicting performance on three different levels of symbolic approximate arithmetic ability.

	Predictors	Model 1											
		Level 1				Level 2				Level 3			
		B (*SE*)	β	*R^2^*	*ΔR^2^*	B (*SE*)	β	*R^2^*	*ΔR^2^*	B (*SE*)	β	*R^2^*	*ΔR^2^*
Step 1	Gender	-0.05 (0.04)	-0.12			-0.03 (0.04)	-0.08			-0.06 (0.04)	-0.16		
	Age	-0.15 (0.06)*	-0.25*	*8.4%*	*8.4%*	-0.20 (0.07)**	-0.30**	*10.2%*	*10.2%*	-0.15 (0.06)*	-0.24*	*8.8%*	*8.8%*
Step 2	Gender	-0.01 (0.04)	-0.03			0.02 (0.04)	0.05			-0.01 (0.04)	-0.03		
	Age	-0.13 (0.06)*	-0.21*			-0.16 (0.07)*	-0.24*			-0.11 (0.06)	-0.18		
	Number line estimation	0.01 (0.002)**	0.30**	*16.1%*	*7.7%*	0.01 (0.003)***	0.40***	*24.0%*	*13.8%*	0.01 (0.002)***	0.40***	*22.9%*	*14.1%*
Step 3	Gender	-0.02 (0.04)	-0.06			0.003 (0.04)	0.008			-0.02 (0.04)	-0.06		

		**Model 2**											

	Age	-0.09 (0.06)	-0.14			-0.11 (0.07)	-0.17			-0.08 (0.06)	-0.12		
	Number line estimation	0.004 (0.003)	0.18			0.01 (0.003)*	0.27*			0.01 (0.003)*	0.30*		
	Number comparison	0.23 (0.11)*	0.25*	*20.3%*	*4.2%*	0.28 (0.12)*	0.27*	*28.9%*	*4.9%*	0.20 (0.11)	0.21	*26.0%*	*3%*
Step 1	Gender	-0.05 (0.04)	-0.12			-0.03 (0.04)	-0.08			-0.06 (0.04)	-0.16		
	Age	-0.15 (0.06)*	-0.25*	*8.4%*	*8.4%*	-0.20 (0.07)**	-0.30**	*10.2%*	*10.2%*	-0.15 (0.06)*	-0.24*	*8.8%*	*8.8%*
Step 2	Gender	-0.04 (0.04)	-0.12			-0.03 (0.04)	-0.08			-0.06 (0.04)	-0.15		
	Age	-0.09 (0.06)	-0.14			-0.11 (0.07)	-0.17			-0.07 (0.06)	-0.12		
	Number comparison	0.32 (0.10)**	0.33**	*18.2%*	*9.8%*	0.42 (0.11)***	0.40***	*24,2%*	*14.0%*	0.34 (0.10)**	0.36**	*20.0%*	*11.2%*
Step 3	Gender	-0.02 (0.04)	-0.06			0.003 (0.04)	0.008			-0.02 (0.04)	-0.06		
	Age	-0.09 (0.06)	-0.14			-0.11 (0.07)	-0.17			-0.08 (0.06)	-0.12		
	Number comparison	0.23 (0.11)*	0.25*			0.28 (0.12)*	0.27*			0.20 (0.11)	0.21		
	Number line estimation	0.004 (0.003)	0.18	*20.3%*	*2.1%*	0.01 (0.003)*	0.27*	*28.9%*	*4.7%*	0.01 (0.003)*	0.30*	*26.0%*	*5.9%*

*^∗^*p* < 0.05; ^∗∗^*p* < 0.01; and ^∗∗∗^*p* < 0.001*.

To assess the relative contribution of performance on the two tasks to the 3 levels of difficulty on symbolic approximate arithmetic performance, a second regression model was conducted, reversing the order of entry. Gender and age were entered first, followed by the error rate of number comparison performance and the PAE of number line estimation performance, respectively. For Level 1, number line estimation performance was not a significant predictor of symbolic approximate arithmetic performance after controlling for gender, age, and number comparison performance. For Level 2, the regression coefficient of both number line estimation performance and number comparison performance were significant. For the most difficult level (Level 3), number comparison performance was not a significant predictor after number line estimation performance was entered into the model. For Levels 1 and 2, number comparison performance was still a significant predictor for symbolic approximate arithmetic performance after number line estimation performance was entered into Model 2. However, for Level 3, number comparison performance was not a significant predictor of symbolic approximate arithmetic performance when the number line estimation performance was entered into the model. As symbolic approximate arithmetic performance became more difficult, the *R*-square change uniquely carried by number line estimation performance increased gradually, becoming 2.1% for Level 1, 4.7% for Level 2, and 5.9% for Level 3 after number comparison was controlled. It should be noted that number line estimation performance significantly improved the fit of the model for Levels 2 and 3 of symbolic approximate arithmetic performance (**Table 3**, Model 2).

## Discussion

The current study aimed to investigate the relations between two basic numerical magnitude processing abilities (semantic and spatial) and symbolic approximate arithmetic performance. The results supported the two hypotheses proposed. First, both number magnitude comparison and number line estimation abilities were significantly correlated with the performance on symbolic approximate arithmetic tasks. Second, the relations between the two basic numerical magnitude processing abilities and symbolic approximate arithmetic performance varied with a change in the difficulty of the symbolic approximate arithmetic tasks; with an increase in the difficulty of the symbolic approximate arithmetic task, the contribution of number magnitude comparison ability decreased, whereas the contribution of number line estimation ability increased. The results indicate that number line estimation ability plays a particularly important role in symbolic approximate arithmetic performance with a higher level of difficulty.

### Similarity Between Semantic and Spatial Number Magnitude Processing Abilities

Previous studies have found significant relations between arithmetic ability and number magnitude comparison or number line estimation abilities (Booth and Siegler, 2008; Gunderson et al., 2012; Sasanguie and Reynvoet, 2013; Bartelet et al., 2014), as well as significant correlations between number magnitude comparison ability and number line estimation ability (Laski and Siegler, 2007). In our study, we found that both numerical magnitude tasks correlated with each other and had significant correlations with symbolic approximate arithmetic performance, which was consistent with previous studies (Gilmore et al., 2007; Laski and Siegler, 2007; Gunderson et al., 2012).

Performance on number magnitude comparison and number line estimation tasks may rely on the same underlying representation, similar to a compressed mental number line (Gallistel and Gelman, 1992; Dehaene, 2011). Specifically, a mental number line representation implies that magnitudes are represented as a Gaussian distribution around the true location of each specific number, with partially overlapping representations for nearby numbers. Such a representational organization leads to greater difficulty in discriminating between nearby numbers. This is reflected in both higher error rates and longer reaction times for near distance pairs when compared with far distance ones in a comparison task (distance effect) and in the inaccurate estimation of the location of specific numbers in a number line task within the range of familiar numbers. Because of the common representation, symbolic approximate arithmetic performance is significantly correlated with both basic numerical magnitude processing tasks. And both basic numerical magnitude processing tasks were related to each other.

### Differences Between Semantic and Spatial Number Magnitude Processing Abilities

The results of the current study were consistent with previous studies that demonstrated that number comparison and number line estimation abilities play different roles in arithmetic performance with different levels of difficulty (Sasanguie and Reynvoet, 2013; Linsen et al., 2014).

The different contributions of the two basic numerical magnitude processes to symbolic approximate arithmetic performance could be explained by the evidence provided for the dissociation between number comparison and number line estimation abilities. For example, a patient who had damage to the left posterior parietal lobe was impaired in the ability to process the relative positions of numbers, while the ability to perform tasks that required the processing of the meaning of numerical magnitude was preserved (Turconi and Seron, 2002). In addition, functional magnetic resonance imaging (fMRI) studies and event-related potential (ERP) studies showed separate neural circuits or brain signatures for processing numerical magnitude information and numerical spatial information. Researchers have found the different spatial and temporal courses between numerical processing and ordinal processing using ERPs (Turconi et al., 2004; Rubinsten et al., 2013). Using fMRI, researchers have also found that the ordinal processing and cardinal number processing have a separate brain activation in the intraparietal sulcus (Tang et al., 2008). Furthermore, a behavioral study failed to find a transfer effect between number comparison and number line estimation abilities (Maertens et al., 2016).

### Difficulty of Symbolic Approximate Arithmetic Performance and Number Magnitude Processing

The current study found that when the difficulty of symbolic approximate arithmetic tasks increased, the number line estimation ability contributed more to the symbolic approximate arithmetic performance, whereas the number comparison ability contributed less.

One possible explanation is that the numerical magnitude comparison ability develops earlier than the number line estimation ability. This hypothesis is supported by a variety of findings. First, studies in developmental psychology have shown that the ability to process quantities is part of a “cognitive core knowledge,” recent studies have found that accuracy on a symbolic number comparison task in the range of 1–100 reaches about 90% in 6-year-old kindergartners (Kolkman et al., 2013). However, their performance on number line estimation tasks in the same numerical range continues to show sustained development across grades 1–3 (Booth and Siegler, 2006). Second, a developmental model of number acquisition (Von Aster and Shalev, 2007) has described the development of numerical cognition in four steps with the learning of the basic meaning of numbers as the first step, the verbal learning of number words as the second step, the connection between the Arabic number system and the former two steps as the third step, and the numerical spatial representation that develops during the school period as a result of the development of the first three steps as the fourth step. Altogether, this evidence suggests that the numerical comparison ability develops earlier than the number line estimation ability. According to the cognitive load theory (CLT), if the extraneous cognitive load (corresponding to symbolic approximate arithmetic performance in our study) was not high, automated schema in long-term memory (corresponding to number semantic processing ability) would be used to solve the problem; whereas if the extraneous cognitive load was high, a complex schema (corresponding to number spatial processing ability) should be developed to solve the complex problems (Sweller et al., 1998; Van Merrienboer and Sweller, 2005). Because number semantic processing ability develops earlier than number spatial processing ability, number semantic processing ability should develop earlier in the cognitive process. When the symbolic approximate arithmetic task was simple (the extraneous cognitive load is low), the children’s number comparison ability (automated schema) was used first, whereas when the symbolic approximate arithmetic task became more difficult, the number line estimation ability (developed schema) gradually began to operate.

The second possible explanation is that complex mathematical problems depend on spatial processing ability when compared with simple problems. Behavioral and neuroimaging studies have found that as the difficulty of the mathematical problem increases, spatial ability plays a more significant role. A developmental study (Sasanguie et al., 2012) found that when mathematical ability was tested with complex problems, the number line estimation ability predicted performance more strongly than the number comparison ability. Previous neuroimaging studies for children have demonstrated that complex arithmetic problem activates the parietal lobe more than simple arithmetic problem (Menon et al., 2000; De Smedt et al., 2011; Ashkenazi et al., 2012; Berteletti et al., 2015). Moreover, the number line estimation ability has significant correlations with complex arithmetic performance in the brain. One neuroimaging study showed that number line estimation ability was related to arithmetic performance by comparing the activation of the parietal lobe for simple and complex arithmetic problems (Berteletti et al., 2015). A training study showed that less activation occurred in the parietal lobe in response to a number task following number line estimation training (Kucian et al., 2011). Spatial information always depends on the parietal lobe (see Zacks, 2008, for a meta-analysis and review). Spatial attention and visuospatial working memory abilities were needed to solve the complex arithmetic problems when compared with the simple ones (Zago et al., 2001; Zago and Tzourio-Mazoyer, 2002; Berteletti et al., 2015). In this study, with the increase in difficulty, the sum of the problem was closer to the comparison number. Participants had to rely on much more spatial attention and visuospatial working memory process to retrieve the approximate answers from the mental number line (Knops et al., 2009).

A limitation of the present study is given by the consideration of the SES in which this sample reflected. Specifically, recent findings indicated that SES backgrounds can affect children’s performance on the symbolic approximate arithmetic tasks, suggesting that those from middle-to-high SES backgrounds performed significantly better than age-matched peers from low SES backgrounds (McNeil et al., 2011). Our data were all collected from kindergartens in urban areas, which were assumed to be representatives of middle-to-high SES backgrounds, thereby the question of whether this result will also generalize to other samples remains to be further investigated.

The current study provides new insights into the cognitive mechanisms of symbolic approximate arithmetic performance for kindergartners. The finding that symbolic approximate arithmetic ability is related to basic numerical magnitude processing implies that performance on symbolic approximate arithmetic tasks may be improved through basic number magnitude processing training. The results also suggest that for complex arithmetic tasks, number spatial ability may be more essential. Training studies have found that spatial representation of numbers could be taught using games (Siegler and Ramani, 2008; Kucian et al., 2011; De Smedt et al., 2013). These studies used computer games or board games to teach the spatial presentation of numbers. Feedback, provided in the game guides, helps children to learn the correct position of numbers. Future studies should be conducted to explore the effects of such game training on different levels of arithmetic performance, as well as on exact arithmetic ability in the same study.

The participants in this study were Chinese kindergartners. Previous cross-cultural studies had found that Chinese kindergartners had superiority in exact arithmetic ability (Rodic et al., 2015) and number line estimation ability (Siegler and Mu, 2008). This superiority could be because of the base-10 structure system of number name which could help Chinese kindergartners to count and understand the meaning of numbers (Miller et al., 1995). Secondly, Chinese children have more information related to numbers in daily life (Kelly et al., 1999). For example, Chinese people use numbers to name months, that is, January in Chinese is “the first month,” February is “the second month,” and so on. Chinese parents typically have higher expectations regarding mathematical achievement when compared with western parents, which influences the Chinese parents to teach their children mathematics at home before entering primary school (see Ng and Rao, 2010, for a review). Further studies could be carried out to investigate if the cultural differences would influence the performance of symbolic approximate arithmetic tasks.

## Author Contributions

WW designed the experiments and drafted the manuscript. WD collected and analyzed the data and drafted the manuscript. CC collected the data. JH and JQ provided methodological advice. YK revised the manuscript.

## Conflict of Interest Statement

The authors declare that the research was conducted in the absence of any commercial or financial relationships that could be construed as a potential conflict of interest.
